# Increased prefrontal activity and reduced motor cortex activity during imagined eccentric compared to concentric muscle actions

**DOI:** 10.3389/fnhum.2012.00255

**Published:** 2012-09-07

**Authors:** C.-J. Olsson, M. Hedlund, P. Sojka, R. Lundström, B. Lindström

**Affiliations:** ^1^Centre for Population Studies, Ageing and Living Conditions, Umeå UniversityUmeå, Sweden; ^2^Umeå Centre for Functional Brain Imaging, Umeå UniversityUmeå, Sweden; ^3^Department of Community Medicine and Rehabilitation, Umeå UniversityUmeå, Sweden; ^4^Department of Health Sciences, Mid-Sweden UniversityÖstersund, Sweden; ^5^Department of Public Health and Clinical Medicine, Occupational Medicine, Umeå UniversityUmeå, Sweden

**Keywords:** motor imagery, fMRI, eccentric, concentric, force modulation

## Abstract

In this study we used functional magnetic resonance imaging (fMRI) to examine differences in recruited brain regions during the concentric and the eccentric phase of an imagined maximum resistance training task of the elbow flexors in healthy young subjects. The results showed that during the eccentric phase, pre-frontal cortex (BA44) bilaterally was recruited when contrasted to the concentric phase. During the concentric phase, however, the motor and pre-motor cortex (BA 4/6) was recruited when contrasted to the eccentric phase. Interestingly, the brain activity of this region was reduced, when compared to the mean activity of the session, during the eccentric phase. Thus, the neural mechanisms governing imagined concentric and eccentric contractions appear to differ. We propose that the recruitment of the pre-frontal cortex is due to an increased demand of regulating force during the eccentric phase. Moreover, it is possible that the inability to fully activate a muscle during eccentric contractions may partly be explained by a reduction of activity in the motor and pre-motor cortex.

## Introduction

It has long been a question, what underlies the inability to fully activate a muscle during a maximum lengthening (eccentric) muscle action (Enoka, 1996). It is a well known phenomenon that for the same level of force, sub-maximum shortening (concentric), and lengthening (eccentric) contractions display different physiological characteristics. For example, concentric and eccentric contractions have different EMG patterns (Bigland and Lippold, 1954; McHugh et al., 2002), with lower amplitude during the eccentric contraction. It is also known that the force achieved during a maximum eccentric contraction is greater than during a maximum concentric contraction (Katz, 1939) due to the properties of the cross-bridges (Rack and Westbury, 1974). However, there seems to be fewer motor units recruited during maximum eccentric contractions compared to concentric contractions (Moritani et al., 1988) as well as a lower discharge rate of these units (Pasquet et al., 2006). Some studies have even proposed that concentric and eccentric contractions display different recruitment order of the motor units (Howell et al., 1995), whereas other studies reported a recruitment order that is identical for both types of contractions (Søgaard et al., 1996). Thus, even though an exercise that includes both concentric and eccentric contractions, such as during resistance training of elbow flexors, the physiology behind the two contractions appears to differ substantially. Supposedly, these physiological differences have a cortical counterpart that is sending out different commands for the two kinds of contractions. Indeed, it has been proposed that the brain has different control strategies for concentric and eccentric contractions, possibly with inhibitory mechanisms at the central level (Duclay and Martin, 2005; Duchateau and Enoka, 2008) that may be reduced with training (Aagaard et al., 2000). For example, during concentric contractions the cortical excitability is larger compared to eccentric contractions (Abbruzzese et al., 1994) and eccentric contractions are more variable and appear to be more demanding by the central nervous system (Christou and Carlton, 2002; Fang et al., 2004).

Only a few studies have attempted to address the neural control of concentric vs. eccentric contractions. In a series of studies, using imaging methods based on electroencephalogram (EEG), Fang and colleagues (Fang et al., 2001, 2004) showed that eccentric muscle contractions have an earlier onset time in the frontal parts of the brain, as well as a higher cortical signal in relation to preparation of the movement. This was interpreted as if the brain plans and executes eccentric contractions differently than concentric contractions. However, no direct evidence has been presented that can account for the lower amplitude of the EMG signal or the proposed inhibitory mechanism during eccentric contractions.

One explanation for why so few studies have been performed on this topic may be related to methodological issues. In most imaging methods (e.g., functional magnetic resonance imaging, fMRI) it is difficult to actually produce maximum contractions within the scanner because of difficulties having equipment in the magnetic field. Simple movements, such as wrist flexions, are easily made. However, maximum contractions are more difficult as well as more likely to result in movements of the head, which then can lead to poor quality of the data. Nevertheless, only by studying maximum contractions we will understand the neuronal system that inhibits muscular activation that one cannot overcome despite maximum volitional effort. During sub-maximum contractions the idea is to reduce muscular activation in order to meet task requirements, and thus should not be used to this purpose. Recently though, Guillot et al. (2007) showed that when simulating eccentric and concentric contractions using motor imagery a similar EMG pattern, yet lower in amplitude, appeared as during executed concentric and eccentric contractions. Over the years motor imagery has been a legitimate approach to study motor representations because of the similarities between imagined and executed motor tasks (Jeannerod, 1995; de Lange et al., 2008; Olsson et al., 2008; Olsson and Nyberg, 2010). Moreover, studies have shown that imagery can be used to improve strength related tasks (Ranganathan et al., 2004), but it is still a controversial issue with large individual differences. Although, one must also remember that there are differences between motor imagery and execution, and it has been shown that there may be partially different activation patterns within the motor system during motor imagery and execution (Gerardin et al., 2000). Moreover, recent studies have shown that physical experience shapes the underlying neural networks of motor imagery (Olsson and Nyberg, 2011; Olsson, 2012) indicating that in order to fully understand how the brain handles imagined strength tasks experienced and inexperienced lifters should be used. Only then will it be possible to investigate whether potential differences between concentric and eccentric phases are due to lifting experience or a general feature of the human brain. Thus, motor imagery is a method that easily can be performed in a scanner and, therefore can be used as a first attempt to study the underlying neural mechanisms of maximum concentric and eccentric contractions.

In the present study, we used fMRI to investigate differences in recruited brain regions during the concentric phase compared to the eccentric phase when simulating resistance training of the elbow flexors. Based on previous findings that eccentric contractions require more control (Fang et al., 2001), we hypothesized that imagined eccentric contractions should be associated with activation of the prefrontal lobe of the brain compared to imagined concentric contractions. Further, based on the prediction that for eccentric contractions the brain exhibits inhibitory actions (Duclay and Martin, 2005), we hypothesized that the imagined eccentric contractions should be associated with a reduction of activation in the motor and pre-motor cortex. Based on previous studies showing that motor imagery is associated with the actual ability to perform the action physically (Olsson et al., 2008; Olsson and Nyberg, 2011; Olsson, 2012) we believed that there could possibly be differences in recruited brain regions if the participants did not have sufficient physical experience of maximum resistance training of elbow flexors. Therefore, we also hypothesized that there would be differences in recruited brain regions between a group of experienced weight lifters in comparison to a group of weight training novices.

## Methods

### Participants

Eighteen healthy individuals took part of this study (mean age 26.4; 10 females). Participation was voluntarily and all participants gave their informed consent. In order to be able to control for possible task specific experience related effects (Olsson et al., 2008; Olsson and Nyberg, 2010, 2011) 10 of these participants had resistance training experience of both concentric and eccentric training of at least one year (mean age 25.8; five females) and eight were novices of resistance training (mean age 27.3; five females). This study was approved by the Regional Ethical Review Board in Umeå.

### fMRI

#### Imaging parameters

Blood oxygen level dependent (BOLD) T2^*^ images were collected using a gradient echo-planar (EPI) sequence on a 3T scanner (Philips Medical Systems, Netherlands) with the following imaging parameters. TR = 1500 ms; 31 slices acquired: 3.44 × 3.44 mm in-plane × 4.65 mm thick; an eight-channel SENSE head coil with a SENSE-factor of 2.6. Images were analyzed using SPM5 (Wellcome Department of Cognitive Neurology, London, UK). Before the statistical analysis the images were pre-processed using the following steps: realignment and unwarping, slice-timing correction, normalization to MNI space (Montreal Neurological Institute), and smoothing (8.0 mm FWHM Gaussian kernel). Temporal autocorrelations were estimated using an AR-1 model and images were high-pass filtered (128 second cut-off).

#### Procedure

**Figure 1:**
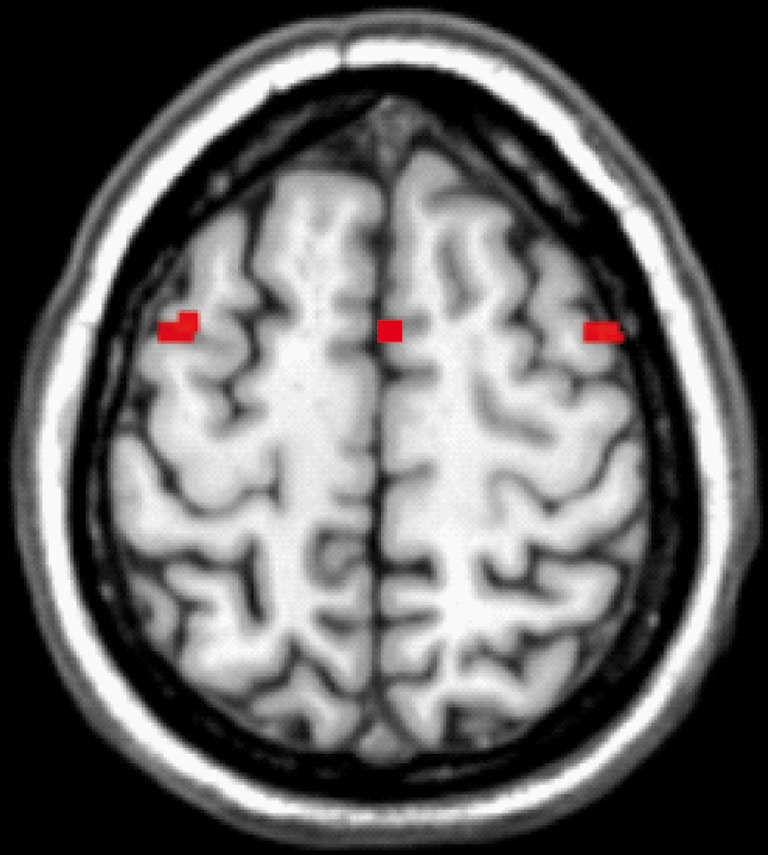
**Jointly recruited brain regions following a conjunction analysis between imagined and executed sub-maximum contractions of the elbow flexors (*p* < 0.00001 FWE corrected).** The recruited brain regions were in pre-motor cortex: *x*, *y*, *z* = 44, 4, 58, *k* = 19, *T* = 7.85; −38, 4, 56, *k* = 22, *T* = 7.72 and in the supplementary motor cortex (SMA): *x*, *y*, *z* = 8, 10, 64, *k* = 29, *T* = 7.62. This indicates that imagery and executed sub-maximum contractions of the elbow flexors share common motor regions and thus we are able to address the research question of eccentric vs. concentric maximum contractions using motor imagery. Note: we also performed separate analysis to investigate if there were differences present between imagined and executed contractions. The results from that analysis showed that all regions recruited for the imagined task was present for the executed task. Moreover, for the executed task the *T*-values were in general stronger as well as the extent of voxels were larger. Also, primary motor cortex was recruited during the execution. These findings are in accordance with previous studies (e.g., Ehrsson et al., 2003).

Despite the substantial amount of literature showing overlapping neural networks between imagined and executed actions, elbow joint movements have never been used as a task, although this is a task most people have experienced in life. Therefore, a pilot study was conducted to examine if imagined and performed sub-maximum concentric and eccentric contractions of elbow flexors would recruit similar motor regions of the brain. If so, then it would be likely that an imagined maximum contraction also would be sharing motor regions with an executed maximum contraction, and we should be able to use motor imagery to study maximum contractions since those are difficult to perform inside the MR-scanner. Positioned in the scanner one participant first performed a series of sub-maximum alternating concentric and eccentric contractions of the elbow flexors using both arms. The same task was then repeated, using motor imagery. In order to be able to exclude potential artifacts and to be sure about the strengths of the results from the pilot a conservative threshold was adopted (*p* < 0.00001 FWE) in the analysis. The results from the pilot study are illustrated in Figure 1, which shows overlapping motor regions for imagined and executed sub-maximum contractions. This strengthens the use of motor imagery to study elbow flexion in more detail as well as giving us the opportunity to study possible additional brain regions that would be recruited for maximum contraction and not sub-maximum contractions. None of the data from the pilot was included in the main study since the aim of the pilot was different from that of the main study.

Imagery can be performed from either an external (third person) or internal (first person) perspective. Studies have shown that the internal perspective is more closely related to the actual execution (Jeannerod, 2001) and more appropriate when studying motor tasks (Holmes and Collins, 2001). Before scanning the participants were given instructions about how to use the internal perspective to perform motor imagery. The instruction emphasized that they should imagine from the internal perspective and that they should attempt to feel as if the movement was being executed without actually performing it. The participants were then given a written instruction about how to perform an imaginary resistance-training task of the elbow flexors using both arms. Again, the written explanation of the task emphasized on feeling as if the movement was done from the internal perspective. In the instruction they were also told that the load was so heavy that they had to imagine a maximum contraction for the concentric phase and the eccentric phase, respectively, i.e., the imagined eccentric load was higher then the concentric load. The reason for this is that the eccentric strength is greater than the concentric strength and we wanted to simulate maximum voluntary concentric and eccentric contractions. Thus, on a cognitive level both tasks were similar, that is for both the eccentric phase and the concentric phase the task was to imagine maximum contractions.

**Figure 2:**
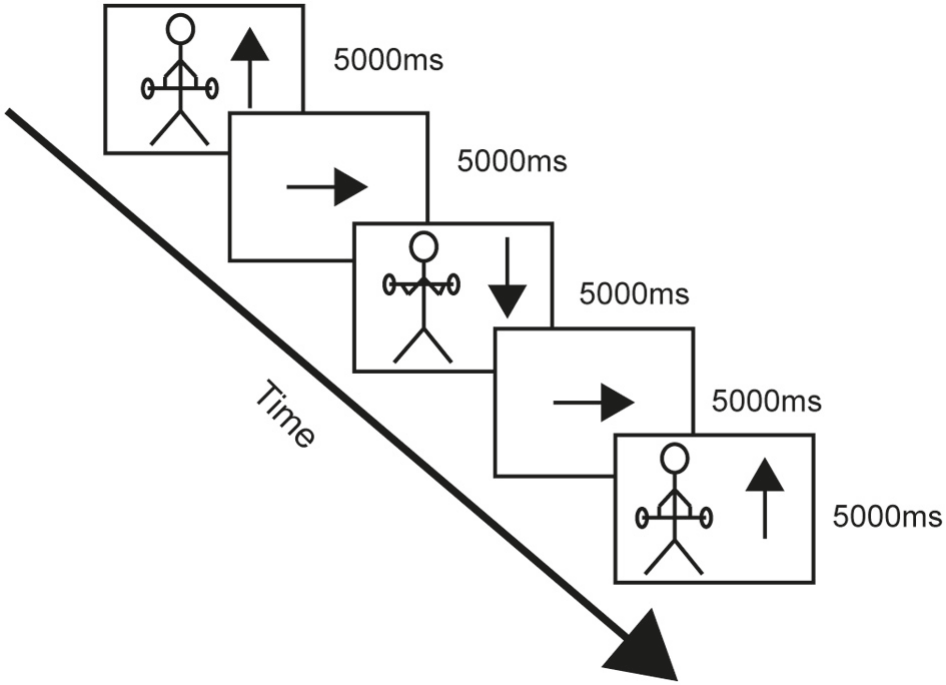
**A schematic drawing of one cycle of the imagined concentric and eccentric contractions**.

When positioned in the scanner the participants performed the task continuously as if they were performing a set of repetitions of alternating concentric and eccentric contractions of the elbow flexors. They always started with the concentric phase followed by the eccentric phase since that is how resistance training of the elbow flexors normally is performed. Inside the scanner there was a tilted mirror attached to the head coil which allowed the participants to see a screen onto which they were given instructions when to start and stop the imagined contractions. Each complete cycle of the task was imagined in the same order each time and was divided into four phases. The start of the task was when the participants saw an up-pointing arrow (↑) on the screen and they started to imagine raising the weight (concentric contraction). Then, when they saw an arrow pointing to the side (→) they rested imagining holding the arms flexed with weight unloaded, until they saw an arrow pointing down (↓) which indicated that they should imagine lowering the weight (eccentric contraction). The cycle ended with an arrow pointing to the side (→) where they rested imagining holding the arms extended with weight unloaded (see Figure 2). The duration of each phase (including the rest) was 5 s, and 18 complete maximum cycles were simulated. Cushions and headphones were used to stabilize the participants and to avoid head movements. After scanning the participants were asked if they had any problems performing the imagery task.

#### Statistical analysis

In this study we used a blocked design, and three conditions (concentric, eccentric, and rest) were set up as separate regressors. Then single subject analyses were made using the general linear model and statistical parametric maps (SPMs) were generated through *t*-statistics. After that, random effects analyses were performed to reveal group averaged data. To first investigate the hypothesis regarding potential group differences between experienced and inexperienced lifters we first compared the group of experienced weight lifters with the group of weight lifting novices by calculating [(eccentric > concentric, for experienced) > (eccentric > concentric, for novices)] and vice versa, as well as [(concentric > eccentric, for experienced) > (concentric > eccentric, for novices)] and the reverse contrast. Because no differences were shown based on the performed analysis, we decided to analyze all participants as one group. Thus, the main contrasts to investigate the hypothesis regarding differences between concentric and eccentric contractions were [concentric > eccentric] and [eccentric > concentric]. For this analysis the threshold level was set to 0.001 uncorrected because of the tight comparison. In order to more clearly understand how the recruited brain regions behaves for the different conditions (concentric or eccentric) BOLD-values for each local maxima identified by the contrasts were extracted. The BOLD values were calculated as the signal percentage change relative the mean of session (beta values) and thus provided more detailed information regarding differences between the two conditions (eccentric and concentric) that was not given by the original contrasts. By extracting these values we will be able to look at the direction of the brain activity. To further substantiate between the two conditions, and for a more detailed analysis we also extracted individual task specific BOLD-values from the entire cluster of the identified brain regions. These values (both peak maxima and cluster) were then analyzed using ANOVAs between conditions. Moreover, to further investigate potential group differences between experienced and novice lifters ANOVAs were conducted for each significant peak of activation from the group analysis, this is important since the limited number of individuals in each group may lead to misinterpretations from the first between group analyses. For the follow up analyses using the extracted BOLD values a standard significance level was used, *p* < 0.05. In order to investigate potential shared regions between the eccentric and concentric conditions a conjunction analysis was made between [eccentric > rest] and [concentric > rest]. For visualization of brain activity a template image was used from MRIcro (www.sph.sc.edu/comd/rorden/mricro.html). For BOLD-plots an in house program (DataZ) was used. Anatomical localizations were determined using the Talairach and Tournoux atlas.

## Results

None of the participants reported any problems with the imagery tasks and followed instructions accordingly. The between group analysis (see “Methods”) revealed no differences between the two groups. All ANOVAs between experienced weight lifters and novices showed non-significant results, thus no differences were present between the two groups (all *p*'s > 0.05). Thus, in the results presented below all participants are included as one group.

**Figure 3:**
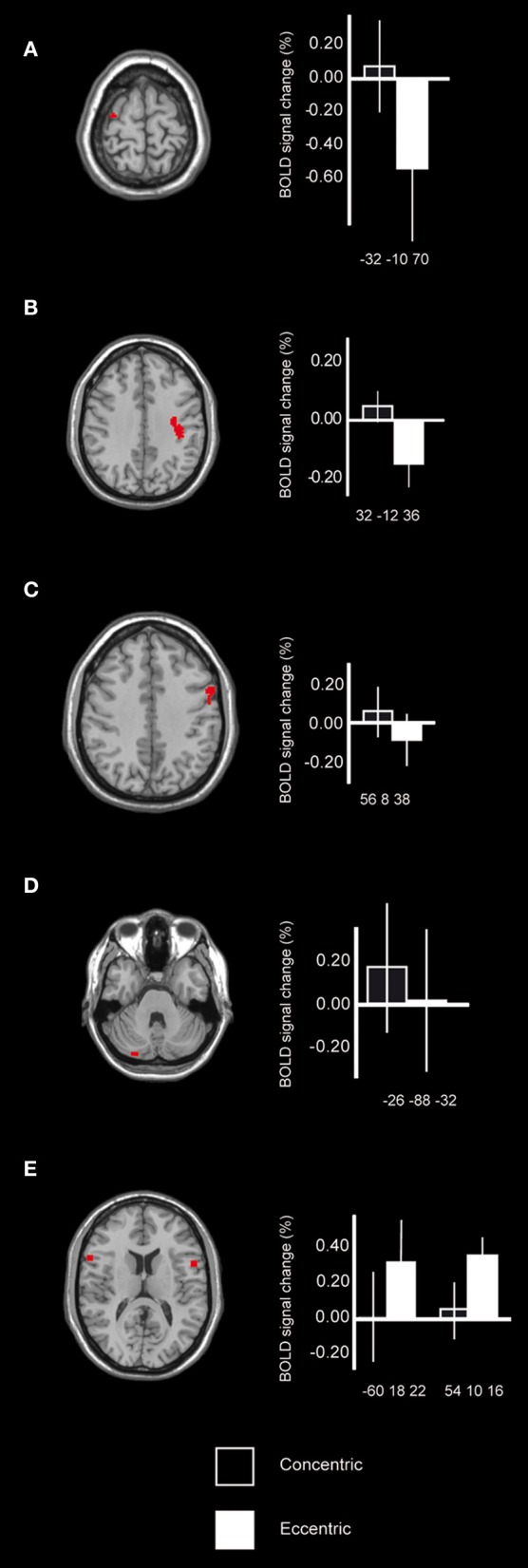
**Cortical activation differences while contrasting simulated maximum concentric and eccentric contractions.** Motor cortex, BA 6/4 **(A,B)** as well as BA 6 **(C)**, showed increased signal change during the simulated concentric contractions in comparison with the simulated eccentric contractions. When comparing the signal change to mean of session it was shown that during the eccentric contractions these regions were instead reduced possibly reflecting a suppressing mechanism involving the cortex. There was also motor recruitment within the cerebellum for the concentric contractions when compared to the eccentric contraction **(D)**. Pre-frontal cortex, BA 44 **(E)** was recruited during the simulated eccentric contractions in comparison with the simulated concentric contractions, possibly reflecting the additional control that is required during eccentric movements and its importance in regulating force. Bars indicate the percentage signal change relative mean of session, error bars are standard error, and coordinates are in MNI-space.

To test the hypothesis regarding differences between the eccentric phase and the concentric phase it was first shown that when comparing brain activation during the concentric phase to the eccentric phase, regions that were recruited were found within the motor system. More specifically, peaks of activation were in the pre-central cortex, *x*, *y*, *z* = −32, −10, 70 [BA 6; *T* = 5.21; *F*_(1, 34)_ = 8.4, *p* < 0.05; *k* = 15] (Figure 3A) and 32, −12, 36, [BA 4/6, the cluster extends from the peak toward BA 4; *T* = 4.79; *F*_(1, 34)_ = 6.3, *p* < 0.05; *k* = 34] (Figure 3B), the pre-motor cortex, BA 6 [*x*, *y*, *z* = 56, 8, 38, *T* = 4.07; *F*_(1, 34)_ = 4.4, *p* < 0.05; *k* = 22] (Figure 3C) and cerebellum bilaterally [*x*, *y*, *z* = −26, −88, −32, *T* = 4.28; *F*_(1, 34)_ = 5.4, *p* < 0.05; *k* = 15; 20, −64, −50, *T* = 3.90; *F*_(1, 34)_ = 5.4, *p* < 0.05; *k* = 8 and 8, −44, −32, *T* = 3.92; *F*_(1, 34)_ = 4.2, *p* < 0.05; *k* = 5] (Figure 3D). Interestingly the BOLD-plots revealed that for the eccentric phase these regions within the motor cortex were reduced (i.e., lower). Further, when comparing brain activation during the eccentric phase to the concentric phase, recruited brain regions were found in the inferior frontal lobe, BA 44, bilaterally. More specifically, peaks of activation were found in *x*, *y*, *z* = 54, 10, 16 [*T* = 6.07; *F*_(1, 34)_ = 7.1, *p* < 0.05; *k* = 18], −60, 18, 22 [*T* = 5.44; *F*_(1, 34)_ = 5.0, *p* < 0.05; *k* = 21] and −58, 8, 4 [*T* = 3.89; *F*_(1, 34)_ = 6.2, *p* < 0.05; *k* = 12] (Figure 3E). All the above reported regions from the main contrasts showed significant differences between eccentric and concentric contractions also regarding the entire cluster, all *F*'s > 4.0. Hence, both peak values and cluster values significantly differed between conditions.

The conjunction analysis revealed shared motor regions between the two conditions; these regions were in the SMA (*x*, *y*, *z* = 6, 8, 62, *T* = 3.79, *k* = 12) and pre-motor cortex (*x*, *y*, *z* = 44, 2, 60, *T* = 4.26, *k* = 8). Both these regions were close to the regions revealed by the pilot study confirming the accuracy of the task.

## Discussion

The purpose of the present study was to examine differences in recruited brain regions during the concentric phase compared to the eccentric phase, when simulating resistance training of the elbow flexors. The results showed that during the eccentric phase of the imagined task, when compared to the concentric phase, more pre-frontal regions were engaged, confirming our hypothesis (i.e., imagined eccentric contractions should be associated with activation of the prefrontal lobe of the brain compared to imagined concentric contractions). In comparison, when the concentric phase was compared to the eccentric phase of the imagined task, the recruited regions were located in the motor and pre-motor cortex. In fact, the activation of these regions was reduced during the eccentric phase also confirming our hypothesis. There was a joint activation between the two conditions within the pre-motor cortex and the SMA, close to the location of the pilot study. This was expected because of the shared elements of the eccentric and concentric condition, e.g., the arm. However, the analysis between the conditions revealed several differences. Importantly, these differences were present both when examining the local maxima and when examining the entire clusters. Accordingly, the governing neural control appears to differ between eccentric and concentric contractions. Based on the performed analyses, there was no difference in the recruited brain regions between the group consisting of subjects with long experience of heavy resistance training and the group that had none, or minimal, experience of resistance training, thus against what was hypothesized. This was shown both for the between group contrasts as well as the ANOVAs between the groups. Hence, the physical experience achieved by the daily use of eccentric and concentric movements was sufficient in order to be able to imagine muscle actions using motor imagery. However, the size of the groups was small and thus, potential group differences may have been neglected which should be taken into consideration when interpreting this study.

### Eccentric contractions associated with pre-frontal cortex

The brain regions recruited during imagined eccentric contractions compared to concentric contractions were in the pre-frontal cortex (BA 44) bilaterally. Prefrontal cortex is usually associated with cognition since it is involved in almost all high level cognitive tasks including working memory and episodic memory tasks (Ranganath et al., 2003). Thus, the eccentric phase of a movement could possibly be seen as a cognitive demanding task in which the brain has to solve the problem of e.g., controlling the lowering of a weight without overloading the musculotendinous complex. This is further supported by studies that have shown that eccentric contractions are more difficult to control by subjects compared to concentric contractions (Christou and Carlton, 2002). It should be noted, though, that this difference is not due the two tasks (concentric vs. eccentric) being differently imagined at a cognitive level. That is, in both tasks the participants were to imagine a maximum contraction, thus, the cognitive task demands were the same in respect of always having a maximum load. Nevertheless, it appears as if the eccentric phase may require additional help from the pre-frontal cortex possibly in order to control the movement. However, an alternative interpretation could be based on the study by Dafotakis et al. (2008) showing that when a virtual lesion was induced to this region, using transcranial magnetic stimulation (TMS), the force modulation was disturbed. Hence, this region has also been suggested to be involved in force modulation (see also, Spraker et al., 2009). Moreover, eccentric contractions have previously been associated with an earlier onset time in the frontal lobe (Fang et al., 2001, 2004). How the relationship between the prefrontal cortex and the motor cortex is arranged, in terms of relative timing of the different contractions, during force modulation should be further examined using brain imaging techniques with greater temporal resolution than fMRI, such as EEG or MEG. For maximum concentric contractions the brain simply just needs to exclusively use the motor system to optimize the muscle output, which was reflected by the motor cortex activity, as well as the recruitment of the cerebellum. It has been suggested that purkinje cells within cerebellum fires when modulating force (Frysinger et al., 1984). However, more recent studies (Pasalar et al., 2006; Yamamoto et al., 2007) have questioned this hypothesis suggesting that there are still no strong evidence that cerebellum is associated with force related commands (see also Ebner et al., 2011). The results from the present study suggest that cerebellum is recruited in order to maximize motor output during the concentric phase of the movement. For the eccentric phase, however, we suggest that the force modulating commands are not sent from the cerebellum, but rather from the pre-frontal cortex. In this study we cannot directly address whether the increased frontal activity is due to the direction of force rather than contraction type. That is, if a task would be constructed so that an object is to be pushed with an eccentric contraction and released with a concentric contraction, it is plausible that the concentric contraction would require more motor control and thus engage pre-frontal cortex. However, in the present paper no release was done, indicating that we were investigating the lengthening (eccentric) and shortening (concentric) muscle system. Hence, for lengthening muscle actions in comparison to shortening muscle actions increased activity in the frontal lobe is expected possibly reflecting increased demands of motor control as well as modulating force to avoid muscle damage. It also important to add that in this study we decided to investigate isotonic contractions (i.e., contractions that results in movement). It is also possible to position the arm in a maximal contraction mode without movement (isometric contraction). How this is handled within the cortical system of humans needs to be directly addressed in future studies.

### Force regulating mechanisms

It has been proposed that there are inhibitory mechanisms at the spinal level e.g., mediated by the Golgi tendon organ (Chalmers, 2002) to avoid muscle damage. Hence, if the Golgi tendon organ accounts for a reflexive protective mechanism that e.g., appears during eccentric contractions (Gollhofer et al., 1987), there may also be a reduction of activity at the cortical level, as our results suggest. Potentially, during eccentric contractions less muscular activity is needed for a given force, perhaps fewer signals needs to be fired from the brain to the periphery. It would have been desirable to also measure the EMG activity of the biceps muscle during the imagery contractions. However, due to the situation within the magnetic field this was not possible. Although, several studies (Gandevia et al., 1997; Guillot et al., 2007; Lebon et al., 2008) have shown that imagined movements not only involves closed circuits of the brain, instead one can also expect to obtain EMG activity from the involved muscle during imagery contractions. Moreover, there are studies suggesting that the control mechanism rather acts at the spinal level and not at the cortical level. Gruber et al. (2009), for example, showed that motor evoked potentials (MEP) were lower during lengthening actions compared to isometric. However, as the ratio between MEP and the evoked motor response by electrical stimulation at the cervicomedullary junction (CMEP) increased, they suggested that this was an indication that the inhibition of the signal was primarily at the spinal level (see also Sekiguchi et al., 2001). Similar conclusion was reached by Löscher and Nordlund (2002), on the basis of their observation of unchanged MEPs obtained by TMS when comparing maximum lengthening and shortening elbow flexions. In the present study on the other hand, an increased pre-frontal activation was present together with a reduced activity in the motor and pre-motor cortex during imagined eccentric contractions compared to concentric contractions of the elbow flexors. Thus, our findings extend prior findings to also involve cortical brain regions in the force regulating mechanisms of eccentric contractions (c.f., Fang et al., 2001).

### Limitations and future directions

There are limitations with the use of imaginary contractions instead of real contractions when investigating cortical control of muscle actions. Obviously, we need to perform actual maximum contractions inside the scanner to fully understand the cortical control of eccentric and concentric contractions. When performing motor imagery it has been proposed that the supplementary motor cortex would inhibit the motor cortex to prevent motor execution (Kasess et al., 2008). It is, however, not likely that the reduction found in the present study could be explained by such a mechanism since we contrasted two imagined contractions to each other causing the effect to be canceled out. We also used the internal perspective when the participants were imagining the resistance-training task. It is of course difficult to be certain that the participants indeed performed according to the instruction. However, after scanning was done the participants were asked about the imagery performance, and no participants reported any problems following the instructions. Further, the instructions emphasized that the action was supposed to feel as if it was executed but without movements. Also, the external perspective has been associated with increased activation in the visual cortex (Guillot et al., 2009), no such activation was observed in this study and, hence, it is likely that the participants used the internal perspective during the imagery task. Thus, as a first attempt to address the issue of cortical control of eccentric and concentric muscle actions this study provides some interesting insights that needs to be addressed using executed maximum eccentric and concentric contractions as well as different imaging techniques.

Based on the results of this study we can conclude that there are different neural systems underlying imagined maximum concentric and eccentric contractions. Further, we have provided data showing that the activity of the motor cortex is reduced during imagined lengthening muscle actions. If this also accounts for the attenuation of the EMG activity and the selectively recruited motor units (c.f., Duchateau and Enoka, 2008) as well as operation during contractions executed in real life we are yet to find out. If so, then it would be of interest to also examine whether it is modulated by training or rehabilitation. It is of interest to mention in this context that Aagaard et al. (2000) showed that 14 weeks of heavy resistance training reduced the inhibition of the neuromuscular activation. If this was due to changes in the cortical system or at some other level is still not know. The reduced activation presented in this study also provides us a new viewpoint to explore a pending issue concerning better preservation of eccentric strength in subjects with upper motor neuron lesions (Damiano et al., 2000), which has been suggested to be associated with a lack of motor inhibition during eccentric contractions (Clark et al., 2006).

### Conflict of interest statement

The authors declare that the research was conducted in the absence of any commercial or financial relationships that could be construed as a potential conflict of interest.
